# Supplementary material for: Development and Psychometric Evaluation of the Chinese Version of the Life Skills Scale for Physical Education
Source: Int J Environ Res Public Health. 2022 Apr 27;19(9):5324. doi: 10.3390/ijerph19095324 (PMC9104646; doi:10.3390/ijerph19095324)
Supplement: Supplementary file 1 [file ijerph-19-05324-s001.zip › ijerph-1620352-supplementary.pdf]

Supplementary Materials

Chinese Version of the Life Skills Scale for Physical Education (C-LSSPE)

体育教会了我…	一点也没有				非常多
**团队合作**					
1.在团队/小组中工作表现优异	1	2	3	4	5
2.帮助其他团队/小组成员执行任务	1	2	3	4	5
3.能接受他人的意见来提高自己	1	2	3	4	5
4.为了团队/小组的利益与他人合作	1	2	3	4	5
5.帮助培育团队/小组精神	1	2	3	4	5
6.建议团队/小组成员如何提高他们的表现	1	2	3	4	5
7.为了团队/小组的利益改变自己的表现方式	1	2	3	4	5
**目标设定**					
8.设定目标，以便我可以专注于提高	1	2	3	4	5
9.设定具有挑战性的目标	1	2	3	4	5
10.检查自己目标的进展	1	2	3	4	5
11.设定短期目标以实现长期目标	1	2	3	4	5
12.专注于自己的目标	1	2	3	4	5
13.设定练习目标	1	2	3	4	5
14.设定具体的目标	1	2	3	4	5
**社交技能**					
15.主动开启对话	1	2	3	4	5
16.在各种社交场景中互动	1	2	3	4	5
17.他人没有开口也能主动提供帮助	1	2	3	4	5
18.参加团体活动	1	2	3	4	5
19.保持亲密的友谊	1	2	3	4	5
**问题解决**					
20.能仔细考虑问题	1	2	3	4	5
21.比较每个可能的解决方案，以找到最佳选择	1	2	3	4	5
22.为问题创造尽可能多的解决方案	1	2	3	4	5
23.评估问题的解决方案	1	2	3	4	5
**情感技能**					
24.知道如何处理自己的情绪	1	2	3	4	5
25.利用自己的情绪来保持专注	1	2	3	4	5
26.理解当自我情绪化时会表现的不一样	1	2	3	4	5
27.注意自我感受	1	2	3	4	5
**领导能力**					
28.知道如何积极地影响一群人	1	2	3	4	5
29.组织团队/小组成员一起工作	1	2	3	4	5
30.知道如何激励他人	1	2	3	4	5
31.帮助他人解决其表现问题	1	2	3	4	5
32.考虑每个团队/小组成员的个人意见	1	2	3	4	5
33.成为他人的好榜样	1	2	3	4	5
34.为团体/小组设定高标准	1	2	3	4	5

35.认可他人的成就	1	2	3	4	5
**时间管理**					
36.合理安排时间	1	2	3	4	5
37.评估我在每项活动中花费的时间	1	2	3	4	5
38.控制如何使用时间	1	2	3	4	5
39.为高效利用时间设定目标	1	2	3	4	5
**交流能力**					
40.与他人清晰地交谈	1	2	3	4	5
41.注意别人在说什么	1	2	3	4	5
42.注意对方的肢体语言	1	2	3	4	5
43.与他人沟通良好	1	2	3	4	5
